# GnRH Antagonist Protocol With Cessation of Cetrorelix on Trigger Day Improves Embryological Outcomes for Patients With Sufficient Ovarian Reserve

**DOI:** 10.3389/fendo.2021.758896

**Published:** 2021-10-14

**Authors:** Huihui Xu, Shen Zhao, Xinxing Gao, Xian Wu, Lan Xia, Dan Zhang, Jian Li, Aijun Zhang, Bufang Xu

**Affiliations:** ^1^ Department of Obstetrics and Gynecology, Ruijin Hospital, Shanghai Jiao Tong University School of Medicine, Shanghai, China; ^2^ Clinical Research Center, Ruijin Hospital, Shanghai Jiao Tong University School of Medicine, Shanghai, China; ^3^ Department of Histo-Embryology, Genetics and Developmental Biology, Shanghai Jiao Tong University School of Medicine, Shanghai Key Laboratory of Reproductive Medicine, Shanghai, China

**Keywords:** GnRH-ant protocol, cetrorelix, trigger day, embryological outcomes, clinical outcomes

## Abstract

**Objective:**

To evaluate the efficiency and validity of cessation of cetrorelix on trigger day during gonadotropin releasing hormone antagonist (GnRH-ant)-controlled ovarian stimulation of *in vitro* fertilization (IVF) cycles.

**Methods:**

In this retrospective study, a total of 1271 patients undergoing initial IVF cycles following the GnRH-ant protocol were enrolled; 832 patients received cetrorelix on trigger day (Group A) and 439 patients ceased cetrorelix on trigger day (Group B). We compared demographic characteristics, embryological and clinical outcomes between the two groups. A Poisson regression model was used to identify factors that significantly affected embryological outcomes. Patients were further divided into subgroups according to anti-Mullerian hormone (AMH) and age, to assess associations between ceasing cetrorelix on trigger day and embryological outcomes.

**Results:**

There was a significant improvement on embryological outcomes in patients who ceased cetrorelix on trigger day, and there were no significant differences in clinical outcomes or preovulation rates between the two groups. Furthermore, for patients with 1.1 ≤ AMH ≤ 4.7 ng/ml, all embryological outcomes were significantly higher in Group B compared with Group A. For patients with AMH > 4.7 ng/ml, the number of oocytes retrieved, fertilization rate (2PN) of IVF cycles and proportion of day 3 good quality embryos were all significantly higher in Group B. For patients with age < 35 years, all the embryological outcomes, besides the number of available embryos, were significantly higher in Group B than in Group A. There were no differences in embryological outcomes between the two groups when patients were stratified based on age > 35 years or AMH < 1.1 ng/ml.

**Conclusion:**

GnRH-ant protocol with cessation of cetrorelix on trigger day improved embryological outcomes for young patients or patients with sufficient ovarian reserve, and was effective at preventing preovulation.

## Introduction

Gonadotropin releasing hormone (GnRH) antagonist (GnRH-ant) protocol is effective in preventing pituitary luteinizing hormone (LH) surge during controlled ovarian stimulation (COS) for *in vitro* fertilization (IVF) (1). The GnRH-ant protocol avoids many side-effects associated with GnRH agonist (GnRH-a) regimens (flare-ups, hypo-estrogenic, long down-regulation period), and results in a lower incidence of patients developing severe ovarian hyperstimulation syndrome (OHSS) (2). Therefore, this protocol is widely used as a convenient and cost-effective treatment for patients undergoing IVF. However, some studies have reported that the GnRH-ant protocol negatively affected the receptivity of the endometrium (3–6). Other studies have reported a decreased number of oocytes were retrieved following the GnRH-ant protocol, compared with the GnRH-a protocol (7–9). These considerations have limited the application of GnRH-ant protocol in clinical practice.

According to the fixed GnRH-ant protocol, cetrorelix was administered starting day 6 of the simulation cycle with a fixed dose of 0.25 mg per day until the trigger day (10). While on the trigger day, human chorionic gonadotropin (hCG) or short-acting GnRHa was administrated to achieve final oocyte maturation, and oocyte aspiration was performed 35-36 hours later. Cetrorelix was no longer needed during the interval time from trigger to oocyte aspiration, it was safe enough to prevents LH surge during this time (1). In recent years, the GnRH-ant protocol continues to be optimized to improve IVF outcomes. For example, improvements to the protocol have resulted from shorting the duration or reducing dosage of antagonist during COS, compared to the fixed GnRH-ant protocol (11–13). Our previous study established that a flexible low-dose GnRH-ant protocol was as effective as the full-dose fixed protocol for patients with normal ovarian reserve (14). In two small studies, omitting cetrorelix on trigger day reduced the total dose of antagonist without compromising side-effects on embryo quality and pregnancy outcomes (15, 16). These results suggested that it is feasible to reduce the dosage or time of antagonist appropriately. During clinical IVF, based on the long terminal half-life (30 hours) of cetrorelix when injected subcutaneously, and the prolonged time (≥ 20 hours) to clear cetrorelix from the plasma when cetrorelix is administered in multiple doses (17), we made efforts to omit cetrorelix on trigger day for some patients whose LH levels were below 10 IU/L during COS of the fixed full dose GnRH-ant protocol. In the present study, we retrospectively analyzed 1271 patients who underwent the GnRH-ant protocol and either omitted cetrorelix on trigger day or continued cetrorelix on trigger day, to evaluate the safety and compare the outcomes of the two different protocols.

## Materials and Methods

### Patients

The retrospective study was performed at the Reproductive Medical Center of Ruijin Hospital from June 2015 to March 2020. The study protocol was approved by Institutional Ethics Committee of Ruijin Hospital. A total of 1731 patients referred to the reproductive center who underwent their first ovarian stimulation following the GnRH-ant protocol were selected for eligibility in this study. The inclusion criteria were as follows: (a) 20-40 years old, (b) signed informed consent. The exclusion criteria were as follows: (a) LH > 10 IU/L during COS, which indicates the patient was prone to premature LH surge (18), (b) known chromosomal aberration among the parents, (c) endometriosis, (d) adenomyosis, (e) submucosal myoma, (f) intramural myoma close to endometrium or over 5 cm in size. In all, 460 patients were excluded due to the exclusion criteria.

**Figure 1:**
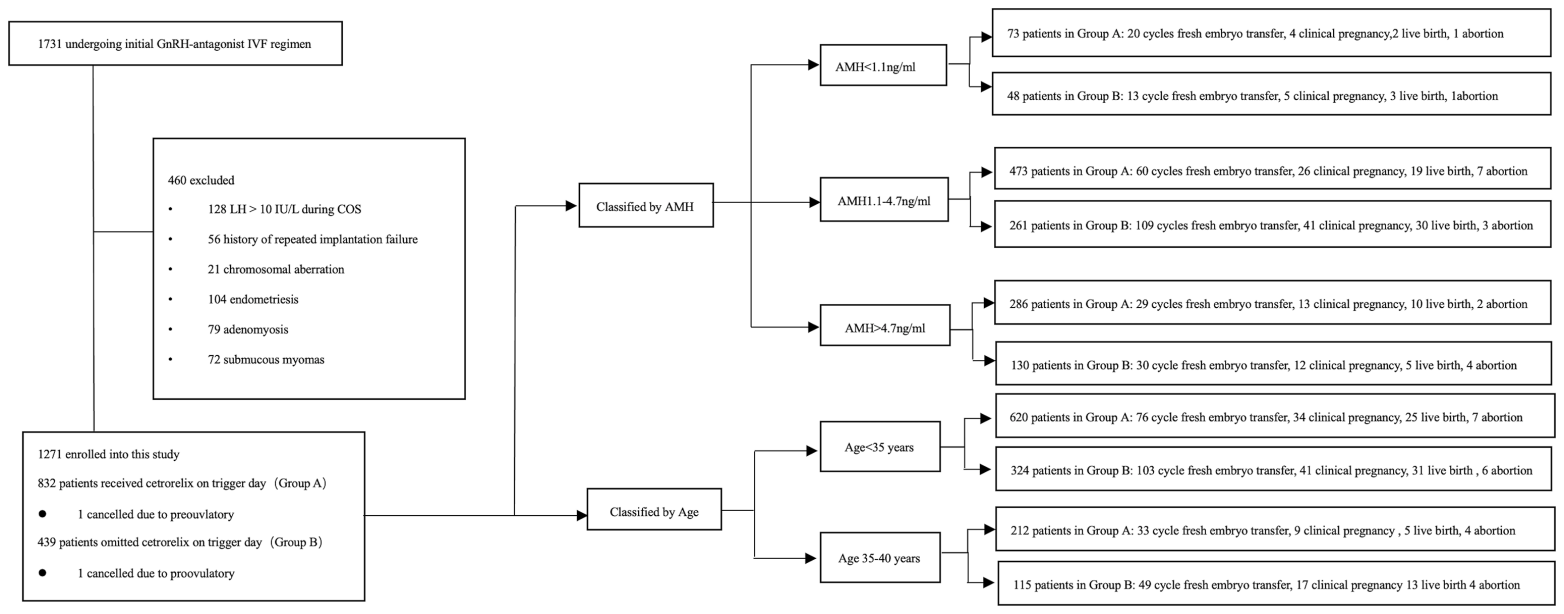
Flowchart of this study.

A total of 1271 patients were enrolled into this study. Patients were divided into two groups according to the administration of cetrorelix, that is, 832 patients received cetrorelix daily until the trigger day (Group A) and 439 patients received cetrorelix daily, ceasing cetrorelix on the trigger day (Group B). The subgroups were divided either according to the AMH or age (19–22). Of these patients, 109 patients in Group A and 152 patients in Group B received fresh embryo transfer (See **Figure 1**).

### Stimulation Protocol

Patients’ baseline FSH, LH, E_2_, P4 levels and antral follicle counts (AFC) were measured on menstrual day 2 of the stimulation cycle. On the same day, recombinant FSH (Gonal-F, Merck-Serono SA, Switzerland) was administered, according to the baseline characteristics; doses ranged from 112.5 to 300 IU per day. Ovarian response was monitored by routine serum FSH, LH, E_2_, P4 levels and follicle scanning every 2 or 3 days during COS. Cetrorelix acetate (0.25mg per day, Cetrotide, Merck-Serono, SA, Switzerland) was administered starting day 6 of the stimulation cycle (14, 23). Patients in Group A received cetrorelix daily through trigger day, and patients in Group B ceased cetrorelix on trigger day. The criteria whether the patient administered the antagonist or not on the day of triggering was determined according to the administering clinician’s usual practice. The final oocyte maturation trigger was administered with either 5000-7000u hCG (Lizhu, Zhuhai, China) or 0.2 mg GnRH agonist (triptorelin acetate; France) when three follicles reached a mean diameter of 17 mm. Oocyte aspiration was performed 35-36 hours after the trigger day.

### IVF and Embryo Quality Assessment

Oocytes were fertilized on the day of oocyte aspiration, fertilization was assessed approximately 16-18 hours after insemination, normal fertilization was confirmed by the presence of 2 pronuclei (PN). Sequential media (Vitrolife, Sweden) was used to culture embryos, and all fertilized oocytes were incubated at an atmosphere of 6% CO2, 5% O_2_ and 89% N_2_ at 37°C. Cleavage stage embryos at day 3 were assessed based on morphological characteristics using a standardized scoring system, including A) the number of blastomeres (BL), (score 2 = five BL, score 3 = six to seven BL and score 4 = eight to ten BL); B) the degree of fragmentation (FR) (score 2 indicates 11-25% fragmentation, score 3 indicates ≥ 10% fragmentation, score 4 indicates no fragmentation; C) the equality or variation in the size of BL, score 1 indicates uniform BL size, and score 0 indicates varying BL size (24). An embryo with score ≥5 was considered as an available embryo, score ≥ 8 was considered as a good quality embryo, and the embryo with score ≥ 9 was considered as a top-quality embryo (24, 25). Blastocyst stage embryos were assessed using the Gardner grading system (26), including both the degree of blastocoel expansion and the quality and cell number of two cell lineages (inner cell mass and trophectoderm). A blastocyst with a score ≥ 3BC was considered as an available blastocyst, and a blastocyst with score ≥ BB was considered to be a top-quality blastocyst (26). The total number of available embryos refers to both the day 3 embryos and blastocysts. Maturation rate was calculated as the percentage of metaphase II oocytes among the total number of oocytes in intracytoplasmic sperm injection (ICSI) cycles. Fertilization rate (2PN) for IVF cycles was calculated as the percentage of normal fertilized oocytes (2PN) of the inseminated oocytes. Fertilization rate (2PN) for ICSI cycles was calculated as the percentage of normal fertilized oocytes (2PN) among MII oocytes. The proportion of day 3 good quality embryos was calculated as the percentage of embryos with score ≥ 8 among the normal fertilized oocytes.

### Embryo Transfer

One or two day 3 embryos with a score ≥ 5 were transferred on day 3, or blastocysts with available ranking were transferred on day 5, following ovum retrieval. For patients with history of caesarean-section, one blastocyst was transferred during the cycle. The freeze-all strategy was implemented for patients diagnosed with moderate to serious OHSS, when the patients received the agonist trigger (27), when a patients’ P4 level was higher than 2 ng/ml on trigger day (28), or when a patient rejected the fresh embryo transfer during the cycle. Transabdominal ultrasound was performed during the transfer procedure. Embryos were loaded into a Cook embryo transfer catheter (Cook IVF, Australia) by an embryologist. The luteal phase was supported by 90 mg of sustained-release progesterone gel (8% Crinone; Merck-Serono, Switzerland), which was administered vaginally starting on the first day after oocyte retrieval. A serum β-HCG test was performed 11 days following transfer of day 3 embryos or 9 days following transfer of blastocyst stage embryos. Clinical pregnancy was defined as visualization of a gestational sac and fetal cardiac activity on transvaginal ultrasound 6 weeks after embryo transfer.

### Statistical Analysis

Continuous data are reported as mean ± SD, and categorical data are presented as frequencies and percentages. Mann-Whitney U tests and Student’s t tests were used to compare means for continuous data. Chi-square tests or Fisher’s exact tests were used to determine the differences between percentages for categorical data. SPSS 25.0 software was used for statistical analysis. A P-value of *p* < 0.05 was considered statistically significant.

The adjusted ORs and 95% confidence intervals (CIs) were calculated by Poisson regression. A two-sided α of less than 0.05 was considered statistically significant. SAS software (V. 9.4) (SAS Institute Inc., USA) was used for additional statistical analysis.

## Results

**Table 1** presents the demographics and clinical characteristics during COS for the 1271 patients. There were no significant differences between the two groups in terms of age, BMI, basal FSH, basal LH, basal E_2_, basal P4, AFC and the causes of infertility, except for higher AMH levels in Group A (4.38 ± 3.20 ng/ml vs. 3.91 ± 3.17 ng/ml, *p* < 0.05). Levels of LH, E_2_, and P4 were not significantly different between groups on the start day of cetrorelix administration or on the trigger day (*p* > 0.05). The dose of cetrorelix received was higher (0.88 ± 0.36 mg vs. 0.74 ± 0.36 mg, *p* < 0.001) and there was a longer duration of cetrorelix administration (5.04 ± 1.41 day vs. 3.82 ± 1.44 day, *p* < 0.001) during ovarian stimulation for Group A. The starting dose of gonadotropin (Gn), stimulation duration and dosage of total Gn, as well as the trigger strategies, including hCG or GnRH-a, were not significantly different between the two groups (*p* > 0.05). Each group had one cancelled cycle due to preovulation.

**Table 1:** Demographic and clinical characteristics of the study participants.

Group A (n = 832)		Group B (n = 439)	*p*
**DEMOGRAPHIC CHARACTERISTICS**			
Age (years)	31.51 ± 4.13	31.63 ± 4.20	0.751
BMI (kg/m^2^)	21.84 ± 2.50	22.17 ± 3.22	0.123
AMH (ng/ml)	4.38 ± 3.20	3.91 ± 3.17	0.003
Basal FSH level (IU/L)	7.32 ± 2.17	7.38 ± 2.26	0.479
Basal LH level (IU/L)	4.56 ± 2.37	4.44 ± 2.47	0.122
Basal E_2_ level (pg/ml)	40.75 ± 16.89	40.09 ± 16.73	0.529
Basal P_4_ level (ng/ml)	0.65 ± 0.34	0.66 ± 0.63	0.069
Antral follicle count	11.66 ± 4.32	11.90 ± 4.62	0.229
**INFERTILITY CAUSES**			
Male factor (%)	277/832 (33.2%)	134/439 (30.5%)	0.316
Tubal factor (%)	625/832 (75.1%)	345/439 (78.6%)	0.167
Polycystic ovary syndrome (%)	33/832 (3.97%)	15/439 (3.42%)	0.625
Ovulation disorders (%)	5/832 (0.60%)	5/439 (1.14%)	0.328
Others^#^	52/832 (6.25%)	32/439 (7.29%)	0.478
**OVARIAN STIMULATION**			
Starting dose of Gn (IU)	188.82 ± 51.15	190.69 ± 50.55	0.139
Stimulation duration of Gn (day)	10.06 ± 1.35	10.02 ± 1.54	0.051
Total Gn (IU)	2480.57 ± 636.05	2447.49 ± 727.56	0.073
Duration of Cetrorelix (day)	5.04 ± 1.41	3.82 ± 1.44	<0.001
Total Cetrorelix (mg)	0.88 ± 0.36	0.74 ± 0.36	<0.001
LH level on start day of Cetrorelix (IU/L)	5.81 ± 5.76	5.96 ± 5.94	0.994
E_2_ level on start day of Cetrorelix (pg/ml)	1271.81 ± 1005.40	1259.70 ± 1092.61	0.422
P_4_ level on start day of Cetrorelix (ng/ml)	0.87 ± 0.48	0.88 ± 0.68	0.098
LH level on trigger day (IU/L)	1.91 ± 1.19	1.87 ± 1.39	0.056
E_2_ level on trigger day (pg/ml)	4565.52 ± 2297.81	4462.43 ± 2701.06	0.112
P_4_ level on trigger day (ng/ml)	1.31 ± 0.57	1.38 ± 0.95	0.295
Number of cycles canceled for pre-ovulation	1/832 (0.12%)	1/439 (0.23%)	0.647
Triggered with hCG	597/832 (71.8%)	309/439 (70.4%)	0.608
Triggered with GnRH-a	235/832 (28.2%)	130/439 (29.6%)	0.608

Group A received cetrorelix daily until the trigger day; Group B received cetrorelix daily, ceasing cetrorelix on the trigger day. ^#^Indicates unexplained infertility after 2 or more attempts of intrauterine insemination. Data is expressed as mean ± standard deviation, or number (percentage). BMI, body mass index; AMH, anti-Müllerian hormone.

**Table 2** demonstrates the embryological outcomes and clinical outcomes between the two groups. The Poisson regression analysis showed a significant improvement on all embryological outcomes in Group B, including the number of oocytes retrieved (12.05 ± 5.55 vs 11.32 ± 4.95, *p* < 0.001), the number of fertilized oocytes (9.37 ± 4.37 vs 8.84 ± 4.33, *p* < 0.001), the number of available embryos (4.34 ± 3.18 vs 4.17 ± 2.92, *p* < 0.005), and the number of top-quality embryos (0.73 ± 1.31 vs 0.55 ± 0.97, *p* < 0.05). Furthermore, the maturation rate (86.6% vs 84.2%, *p* < 0.05), the fertilization rate (2PN) (of IVF cycle was 77.3% vs 73.1%, *p* < 0.001, while of ICSI cycle was 83.0% vs 80.4%, *p* > 0.05), and the proportion of day 3 good quality embryos (9.51% vs 7.80%, *p* < 0.05) were also higher in Group B. For patients who underwent fresh embryo transfer, there were no significant differences in the rate of implantation, biochemical pregnancy, clinical pregnancy, ongoing pregnancy, multiple pregnancy, live birth, abortion, or ectopic pregnancy between Group A (n = 109) and Group B (n = 152). In addition, the endometrium thickness on trigger day, the number of embryos transferred, and the score of embryos transferred were all comparable between the two groups (*p* > 0.05).

**Table 2:** Comparison of embryological outcomes and clinical outcomes between the two groups.

	Group A	Group B	*p*
**EMBRYOLOGICAL OUTCOMES**			
Number of oocytes retrieved	11.32 ± 4.95	12.05 ± 5.55	<0.001
Maturation rate (%)^a^	2149/2553 (84.2%)	1277/1475 (86.6%)	0.040
Number of fertilized oocytes	8.84 ± 4.33	9.37 ± 4.73	<0.001
Fertilization rate (2PN) of IVF (%)^b^	5020/6867 (73.1%)	2948/3813 (77.3%)	<0.001
Fertilization rate (2PN) of ICSI (%)^c^	1728/2149 (80.4%)	1060/1277 (83.0%)	0.059
Number of available embryos	4.17 ± 2.92	4.34 ± 3.18	<0.005
Proportion of day 3 embryos (%)^d^	2419/3470 (69.7%)	1235/1812 (68.2%)	
Proportion of blastocysts (%)^e^	1051/3470 (30.3%)	577/181 (31.8%)	
Proportion of day 3 good quality embryos^f^	527/6748 (7.80%)	381/4008 (9.51%)	0.002
Number of top-quality embryos	0.55 ± 0.97	0.73 ± 1.31	0.040
**FRESH EMBRYO TRANSFER**			
Number of cycles transferred	109	152	
Number of embryos transferred	187	269	
Endometrial thickness on trigger day (cm)	1.01 ± 0.18	1.00 ± 0.15	0.673
Average number of embryos transferred	1.72 ± 0.45	1.77 ± 0.42	0.323
Average score of cleavage embryos transferred	7.01 ± 1.05	7.15 ± 1.17	0.310
Embryos transferred at blastocyst stage	4BB*1 4BC*5	4BB*2 4BC*14	
**CLINICAL OUTCOMES**			
Implantation rate (%)	54/187 (28.9%)	69/269 (25.7%)	0.445
Biochemical pregnancy rate (%)	54/109 (49.5%)	72/152 (47.4%)	0.622
Clinical pregnancy rate (%)	43/109 (39.4%)	58/152 (38.2%)	0.833
Ongoing pregnancy rate (%)	33/109 (30.3%)	45/152 (29.6%)	0.907
Multiple pregnancy rate (%)	7/109 (6.42%)	9/152 (5.92%)	0.868
Live birth rate (%)	31/109 (28.4%)	44/152 (28.9%)	0.929
Abortion rate (%)	11/43 (25.6%)	8/58 (13.8%)	0.134
Ectopic pregnancy rate (%)	1/43 (2.33%)	6/58 (10.3%)	0.234

Group A received cetrorelix daily until the trigger day; Group B received cetrorelix daily, ceasing cetrorelix on the trigger day. Data is expressed as mean ± SD, or number (percentage).

a Maturation rate (%) = No. of Metaphase II oocytes/No. of oocytes retrieved in ICSI cycles.

b Fertilization rate (2PN) of IVF (%) = No. of 2PN zygotes/No. of inseminated oocytes.

c Fertilization rate (2PN) of ICSI (%) = No. of 2PN zygotes/No. of microinjected oocytes.

d Proportion of day 3 embryos (%) = No. of the day 3 available embryos (%)/No. of the available embryos.

e Proportion of blastocysts (%) = No. of the available blastocysts (%)/No. of the available embryos.

f Proportion of day 3 good quality embryos (%) = No. of day 3 good quality embryos/No. of 2PN embryos.

**Table 3** shows that the confounding factors of AMH and age affected embryological outcomes significantly. All embryological outcomes were significantly improved in good ovarian reserve patients with AMH > 4.7 ng/ml, compared to patients with normal ovarian reserve (AMH level between 1.1 and 4.7 ng/ml) and patients with poor ovarian reserve (AMH < 1.1 ng/ml) (*p* < 0.05). The embryological outcomes were poorer in advanced age patients (35-40 years) compared to younger patients (age < 35 years) (*p* < 0.05). BMI was not indicated as a significant confounding factor impacting embryological outcomes. There was a higher number of retrieved oocytes in patients with BMI > 25 kg/m^2^ than in patients with BMI < 20 kg/m^2^, which might be due to the higher proportion of polycystic ovary syndrome (PCOS) in patients with BMI > 25kg/m^2^ (12.67% vs 0.76%, *p* < 0.05) (29).

**Table 3:** Poisson regression analysis of factors associated with the embryological outcomes.

	Oocyte Retrieved	Fertilized Oocytes	Available Embryos	Top-quality Embryos
**AMH < 1.1 ng/ml**	6.10 ± 3.45	4.74 ± 3.09	2.13 ± 1.82	0.22 ± 0.54
**1.1 ≤ AMH ≤ 4.7 ng/ml**	10.48 ± 4.38^*^	8.12 ± 3.91^*^	3.65 ± 2.49^*^	0.47 ± 0.92^*^
**AMH > 4.7 ng/ml**	15.09 ± 4.62^*^	11.87 ± 4.09^*^	5.87 ± 3.36^*^	0.98 ± 1.37^*^
**Age < 35 years**	12.43 ± 4.99	9.75 ± 4.37	4.61 ± 3.10	0.69 ± 1.16
**35 ≤ Age ≤ 40 years**	9.08 ± 4.87^†^	6.94 ± 4.12^†^	3.12 ± 2.41^†^	0.37 ± 0.82^†^
**BMI < 20 kg/m^2^**	10.94 ± 4.85^^#^	8.71 ± 4.21	4.00 ± 2.84	0.59 ± 1.05
**20 ≤ BMI ≤ 25 kg/m^2^**	11.64 ± 5.12	9.06 ± 4.40	4.31 ± 3.03	0.62 ± 1.07
**BMI > 25 kg/m^2^**	12.32 ± 5.88^^^	9.39 ± 5.27	4.17 ± 3.18	0.72 ± 1.39

Values are presented as mean ± standard deviation.

^*^, ^†^ and ^^^ signify P < 0.05 for pos-hoc comparison. ^*^ refers to comparison between the group with AMH < 1.1 ng/ml and the group with AMH between 1.1 and 4.7 ng/ml or the group with AMH > 4.7 ng/ml. ^†^ refers to comparison between group with age < 35 years and the group with age between 35 and 40 years. ^^^ refers to comparison between the group with BMI > 25 kg/m^2^ and the group with BMI < 20 kg/m^2^. ^#^ The incidence of polycystic ovarian syndrome (PCOS) in patients with BMI > 25 kg/m^2^ was higher than in patients with BMI lower than 20 kg/m^2^ (12.67% (19/150) vs 0.76% (2/263), p < 0.05). AMH, anti-Müllerian hormone; BMI, body mass index.

We further analyzed the associations between ceasing cetrorelix on trigger day and embryological outcomes. Among patients whose AMH levels ranged between 1.1 and 4.7 ng/ml, the number of oocytes retrieved (11.06 ± 4.73 vs. 10.16 ± 4.15), the number of fertilized oocytes (8.59 ± 4.13 vs. 7.86 ± 3.75), the number of available embryos (3.95 ± 2.66 vs. 3.48 ± 2.38), and the number of top-quality embryos (0.59 ± 1.08 vs. 0.40 ± 0.81) were all significantly higher in Group B compared with Group A (*p* < 0.05). Also, the maturation rate (86.0% vs 81.3%), fertilization rate (2PN) of IVF cycles (78.1% vs 73.2%) and the proportion of day 3 good quality embryos (9.54% vs 7.88%) were significantly higher in Group B compared to Group A (*p* < 0.05, respectively). For patients whose AMH levels were greater than 4.7 ng/ml, the number of oocytes retrieved (16.22 ± 4.75 vs. 14.57 ± 4.47) and the number of fertilized oocytes (12.69 ± 4.17 vs. 11.49 ± 4.01) were both higher in Group B compared to Group A (*p* < 0.05, respectively). Also, the fertilization rate (2PN) of IVF cycles (76.8% vs 73.0%) and the proportion of day 3 good quality embryos (9.63% vs 7.69%) were significantly higher in Group B compared to Group A (*p* < 0.05, respectively). While the increased number of available embryos and top-quality embryos in Group B did not reach statistical significance. There were no significant differences on embryological outcomes for patients whose AMH levels were lower than 1.1 ng/ml between the two groups (**Table 4**).

**Table 4:** Embryological outcomes of the participants based on AMH subgroups.

Embryological Outcomes	Group A	Group B	*p*
**AMH < 1.1ng/ml**	**n = 73**	**n = 48**	
AMH (ng/ml)	0.73 ± 0.27	0.71 ± 0.25	0.509
Number of oocytes retrieved	6.08 ± 3.44	6.13 ± 3.50	0.827
Maturation rate (%)^a^	92/119 (77.3%)	108/138 (78.2%)	0.855
Number of fertilized oocytes	4.82 ± 3.16	4.63 ± 2.99	0.812
Fertilization rate (2PN) of IVF (%)^b^	239/325 (73.5%)	114/156 (73.1%)	0.915
Fertilization rate (2PN) of ICSI (%)^c^	78/92 (84.8%)	93/108 (86.1%)	0.790
Number of available embryos	2.27 ± 1.91	1.92 ± 1.66	0.321
Proportion of day 3 embryos (%)^d^	134/166 (80.7%)	74/92 (80.4%)	
Proportion of blastocysts (%)^e^	32/166 (19.3%)	18/92 (19.6%)	
Proportion of day 3 good quality embryos^f^	26/317 (8.20%)	17/207 (8.21%)	0.997
Number of top-quality embryos	0.22 ± 0.48	0.23 ± 0.63	0.598
**1.1 ≤ AMH ≤ 4.7ng/ml**	**n = 473**	**n = 261**	
AMH (ng/ml)	2.72 ± 0.93	2.59 ± 1.02	0.038
Number of oocytes retrieved	10.16 ± 4.15	11.06 ± 4.73	0.018
Maturation rate (%)^a^	1065/1310 (81.3%)	724/842 (86.0%)	0.005
Number of fertilized oocytes	7.86 ± 3.75	8.59 ± 4.13	0.026
Fertilization rate (2PN) of IVF (%)^b^	2559/3498 (73.2%)	1596/2044 (78.1%)	<0.001
Fertilization rate (2PN) of ICSI (%)^c^	882/1065 (82.8%)	606/724 (83.7%)	0.623
Number of available embryos	3.48 ± 2.38	3.95 ± 2.66	0.037
Proportion of day 3 embryos (%)^d^	1223/1644 (74.4%)	754/1032 (73.1%)	
Proportion of blastocysts (%)^e^	421/1644 (25.6%)	278/1032 (26.9%)	
Proportion of day 3 good quality embryos^f^	271/3441 (7.88%)	210/2202 (9.54%)	0.029
Number of top-quality embryos	0.40 ± 0.81	0.59 ± 1.08	0.020
**AMH > 4.7ng/ml**	**n = 286**	**n =130**	
AMH (ng/ml)	8.06 ± 2.63	7.75 ± 3.11	0.042
Number of oocytes retrieved	14.57 ± 4.47	16.22 ± 4.75	0.002
Maturation rate (%)^a^	992/1124 (88.3%)	445/495 (89.9%)	0.335
Number of fertilized oocytes	11.49 ± 4.01	12.69 ± 4.17	0.006
Fertilization rate (2PN) of IVF (%)^b^	2222/3044 (73.0%)	1238/1613 (76.8%)	0.005
Fertilization rate (2PN) of ICSI (%)^c^	768/992 (77.4%)	361/445 (81.1%)	0.114
Number of available embryos	5.80 ± 3.19	6.00 ± 3.72	0.995
Proportion of day 3 embryos (%)^d^	1062/1660 (64.0%)	481/780 (61.7%)	
Proportion of blastocysts (%)^e^	598/1660 (36.0%)	299/780 (38.3%)	
Proportion of day 3 good quality embryos ^f^	230/2990 (7.69%)	154/1599 (9.63%)	0.024
Number of top-quality embryos	0.88 ± 1.18	1.19 ± 1.71	0.304

Group A received cetrorelix daily until the trigger day; Group B received cetrorelix daily, ceasing cetrorelix on the trigger day. Three subgroups were divided according to AMH values, AMH <1.1 ng/ml, 1.1 ≤ AMH ≤ 4.7 ng/ml, AMH > 4.7 ng/ml. Data is expressed as mean ± SD, or number (percentage). AMH, anti-Müllerian hormone.

a Maturation rate (%) = No. of Metaphase II oocytes/No. of oocytes retrieved in ICSI cycles.

b Fertilization rate (2PN) of IVF (%) = No. of 2PN zygotes in IVF/No. of inseminated oocytes.

c Fertilization rate (2PN) of ICSI (%) = No. of 2PN zygotes in ICSI/No. of microinjected oocytes.

d Proportion of day 3 embryos (%) = No. of the day 3 available embryos/No. of the available embryos.

e Proportion of blastocysts (%) = No. of the available blastocysts/No. of the available embryos.

f Proportion of day 3 good quality embryos (%) = No. of day 3 good quality embryos/No. of 2PN embryos.

Among patients younger than 35, the number of oocytes retrieved (13.05 ± 5.30 vs. 12.11 ± 4.80), the number of fertilized oocytes (10.23 ± 4.59 vs. 9.50 ± 4.23), and the number of top-quality embryos (0.87 ± 1.42 vs. 0.60 ± 1.00) were significantly higher in Group B compared with Group A (*p* < 0.05, respectively). Meanwhile, the maturation rate (88.2% vs 85.5%), fertilization rate (2PN) of IVF cycles (77.8% vs 73.7%) and the proportion of day 3 good quality embryos (9.60% vs 7.90%) were also significantly higher in Group B (*p* < 0.05, respectively). For patients aged between 35 and 40 years, there was no difference on embryological outcomes between the two groups (**Table 5**).

**Table 5:** Embryological outcomes of the participants based on age subgroups.

Embryological Outcomes	Group A	Group B	*p*
**Age < 35 years**	**n = 620**	**n = 324**	
Number of oocytes retrieved	12.11 ± 4.80	13.05 ± 5.30	0.012
Maturation rate (%)^a^	1754/2051 (85.5%)	1031/1169 (88.2%)	0.033
Number of fertilized oocytes	9.50 ± 4.23	10.23 ± 4.59	0.025
Fertilization rate (2PN) of IVF (%)^b^	4022/5459 (73.7%)	2380/3060 (77.8%)	<0.001
Fertilization rate (2PN) of ICSI (%)^c^	1405/1754 (80.1%)	850/1031 (82.4%)	0.128
Number of available embryos	4.51 ± 2.98	4.80 ± 3.33	0.398
Proportion of day 3 embryos (%)^d^	1930/2799 (69.0%)	1032/1556 (66.3%)	
Proportion of blastocysts (%)^e^	869/2799 (31.0%)	524/1556 (33.7%)	
Proportion of day 3 good quality embryos (%)^f^	427/5407 (7.90%)	310/3230 (9.60%)	0.006
Number of top-quality embryos	0.60 ± 1.00	0.87 ± 1.42	0.025
**35 ≤ Age ≤ 40 years**	**n = 212**	**n = 115**	
Number of oocytes retrieved	9.01 ± 4.66	9.21 ± 5.27	0.803
Maturation rate (%)^a^	395/502 (78.7%)	246/306 (80.4%)	0.561
Number of fertilized oocytes	6.92 ± 4.05	6.97 ± 4.27	0.975
Fertilization rate (2PN) of IVF (%)^b^	1018/1408 (72.3%)	568/753 (75.4%)	0.117
Fertilization rate (2PN) of ICSI (%)^c^	323/395 (81.8%)	210/246 (85.4%)	0.237
Number of available embryos	3.17 ± 2.49	3.03 ± 2.26	0.798
Proportion of day 3 embryos (%)^d^	489/671 (72.9%)	277/348 (79.6%)	
Proportion of blastocysts (%)	182/671 (27.1%)	71/348 (20.4%)	
Proportion of day 3 good quality embryos (%)^f^	100/1341 (7.46%)	71/778 (9.13%)	0.174
Number of top-quality embryos	0.39 ± 0.83	0.35 ± 0.81	0.477

Group A received cetrorelix daily until the trigger day; Group B received cetrorelix daily, ceasing cetrorelix on the trigger day. Two subgroups were divided according to age, Age < 35 years and 35 ≤ Age ≤ 40 years. Data is expressed as mean ± SD, or number (percentage).

a Maturation rate (%) = No. of Metaphase II oocytes/No. of oocytes retrieved in ICSI cycles.

b Fertilization rate (2PN) of IVF (%) = No. of 2PN zygotes in IVF/No. of inseminated oocytes.

c Fertilization rate (2PN) of ICSI (%) = No. of 2PN zygotes in ICSI/No. of microinjected oocytes.

d Proportion of day 3 embryos (%) = No. of the day 3 available embryos/No. of the available embryos.

e Proportion of blastocysts (%) = No. of the available blastocysts/No. of the available embryos.

f Proportion of day 3 good quality embryos (%) = No. of day 3 good quality embryos/No. of 2PN embryos.

## Discussion

Currently, there is no unified agreement whether omission of cetrorelix on trigger day could improve outcomes of the GnRH-ant protocol during COS. This study found that ceasing cetrorelix on trigger day improved embryological outcomes significantly, without adding the risk of premature LH surge and preovulation.

Premature LH surge is defined as a LH level ≥ 10 IU/L, and progesterone level ≥ 1.0 ng/ml, which could lead to luteinization and preovulation during COS (30, 31), and is a leading cause of cycle cancellation or failure during assisted conception. The GnRH-ant protocol is effective in preventing premature LH surge during COS, due to its competitive binding with GnRH receptor. Although premature LH surge is harmful to oocyte development and can cause preovulation, a high enough LH level is indispensable not only for the final stages of oocyte cytoplasmic and nuclear maturation, but also for subsequent follicle rupture and ovulation (32, 33). Thus, excessive suppression of LH has been shown to be adverse for follicular development and IVF outcomes (34, 35). Therefore, a reasonable “threshold” for serum LH during folliculogenesis is crucial during IVF. In our study, despite of the higher AMH levels in Group A, there was a significant improvement on the embryological outcomes in Group B, which indicated that discontinuation of cetrorelix on trigger day could be beneficial for oocyte quality and maturation. For the GnRH-ant protocol, the released suppression of LH level on trigger day might potentially improve the intrafollicular microenvironment and enhance oocyte developmental competence.

In this study, only one cancelled cycle for preovulation occurred in each group, indicating that omission cetrorelix on trigger day did not increase the rate of preovulation for patients without premature LH surge (LH < 10 IU/L during COS). As mentioned before, the terminal half-life of cetrorelix is 30 hours after subcutaneous injection, leading to a lasting duration of cetrorelix plasma concentration for longer than 20 hours (17). Furthermore, the plasma concentration of cetrorelix is reduced at a slower rate when a 0.25 mg dose per day of cetrorelix is injected multiple times, compared with a single administration (17). In our study, the average duration of cetrorelix injection was 3.82 ± 1.44 days in Group B. These doses ensured that, for patients without risk of premature LH surge, the cetrorelix levels in the plasma remained abundant to suppress LH levels, even when cetrorelix was omitted on trigger day.

In addition, there were no significant differences in the clinical outcomes between two groups that underwent fresh embryo transfer. There were no significant differences in endometrium thickness, E_2,_ or P4 levels on trigger day, nor in the average number and the average score of embryos transferred. These data suggest that omitting cetrorelix on trigger day might not affect the receptive state of the endometrium significantly, although multiple studies have reported that the GnRH-ant protocol could adversely affect the endometrium receptivity (36–39).

According to the Poisson regression analysis, AMH and age were found to be significant confounding factors that affect embryological outcomes. We found that among patients with normal ovarian reserve with AMH levels between 1.1 and 4.7 ng/ml, all embryological outcomes were significantly improved in Group B compared to Group A. Similarly, for young patients aged < 35 years, although the increased number of available embryos with no significance in Group B, all other embryological outcomes were significantly higher in Group B compared to Group A. These results suggested that, for patients with normal ovarian reserve or young patients, ceasing cetrorelix on trigger day will be beneficial for embryological outcomes. This might be caused by released LH suppression and improved folliculogenesis, which is crucial for the final stage of oocyte maturation and development (32–35). For patients with good ovarian reserve (AMH > 4.7 ng/ml), the number of retrieved and fertilized oocytes, fertilization rate of IVF cycles and proportion of day 3 good quality embryos were all significantly higher in Group B compared with Group A, indicating that the embryological outcomes were improved notably. The number of available embryos and top-quality embryos were also higher, but not significantly, in Group B. This could be explained: first, the incidence of PCOS was higher in patients with AMH > 4.7 ng/ml, and the oocyte quality in those patients might be influenced by endocrine and intra-ovarian paracrine changes in follicular fluid (40); second, compared to patients with AMH levels between 1.1 and 4.7 ng/ml, there might be a higher proportion of immature oocytes obtained in patients with AMH > 4.7 ng/ml (41). However, for patients with AMH < 1.1 ng/ml or aged between 35 and 40 years, ceasing cetrorelix on trigger day had no benefit on embryological outcomes. The quality of oocytes is generally found to be poor in patients with poor ovarian reserve (42–44). Moreover, in advanced age patients, mitochondrial function and energy production ability are also declined, which negatively impact chromosome segregation and oocyte developmental competence (45–47). In these patients, small fluctuations of LH levels before ovulation do not seem to have much impact on oocytes quality.

The limitations in this study were that, first, this was a retrospective cohort study, the number of recruited patients in each group was not comparable, which could potentially affect the differences in embryological outcomes. Second, the sample size for patients who underwent fresh embryo transfer was limited, which could potentially affect clinical outcomes. A randomized controlled trial with a larger sample size will be carried out to ascertain these research findings.

## Conclusion

Due to the potential negative effects on oocytes quality and endometrium receptivity of GnRH-ant, the GnRH-ant protocol continues to be optimized for better IVF outcomes. Although still far from perfect, our findings indicate that the GnRH-ant protocol with cessation of cetrorelix on trigger day improved embryological outcomes in patients with LH < 10 IU/L during COS, and was effective enough to prevent preovulation.

## Data Availability Statement

The original contributions presented in the study are included in the article/**Supplementary Material**. Further inquiries can be directed to the corresponding authors.

## Ethics Statement

The studies involving human participants were reviewed and approved by Institutional Ethics Committee of Ruijin Hospital. The patients/participants provided their written informed consent to participate in this study.

## Author Contributions

BX and AZ conception and design, review and final approval of the version to be published. JL and DZ analyses the data. HX and SZ draft and revise the article. XG, XW, and LX collect the data and analyze the data. All authors contributed to the article and approved the submitted version.

## Funding

This work was funded by the Natural Science Foundation of China (grant numbers: 82071712, 81771656, 81501249, 81873857, 81701439, 81701513), the Shanghai Municipal Education Commission-Gaofeng Clinical Medicine Grant Support (grant number: 20181803), and the Outstanding Youth Medical Talents of Shanghai “Rising Stars of Medical Talent” Youth Development Program (grant number: SHWSRS(2021)_099).

## Conflict of Interest

The authors declare that the research was conducted in the absence of any commercial or financial relationships that could be construed as a potential conflict of interest.

## Publisher’s Note

All claims expressed in this article are solely those of the authors and do not necessarily represent those of their affiliated organizations, or those of the publisher, the editors and the reviewers. Any product that may be evaluated in this article, or claim that may be made by its manufacturer, is not guaranteed or endorsed by the publisher.
